# Neuropeptide Y Plays an Important Role in the Relationship Between Brain Glucose Metabolism and Brown Adipose Tissue Activity in Healthy Adults: A PET/CT Study

**DOI:** 10.3389/fendo.2021.694162

**Published:** 2021-07-09

**Authors:** Qiongyue Zhang, Qing Miao, Yehong Yang, Jiaying Lu, Huiwei Zhang, Yonghao Feng, Wei Wu, Xiaoming Zhu, Boni Xiang, Quanya Sun, Yihui Guan, Yiming Li, Chuantao Zuo, Hongying Ye

**Affiliations:** ^1^ Division of Endocrinology and Metabolism, Department of Internal Medicine, Huashan Hospital, Shanghai Medical College, Fudan University, Shanghai, China; ^2^ Shanghai Key Laboratory of Metabolic Remodeling and Health, Institute of Metabolism & Integrative Biology, Fudan University, Shanghai, China; ^3^ Positron Emission Tomography (PET) Center, Huashan Hospital, Shanghai Medical College, Fudan University, Shanghai, China

**Keywords:** brown adipose tissue, positron-emission tomography and computed tomography, brain glucose metabolism, neuropeptide Y, statistical parametric mapping

## Abstract

**Introduction:**

Brown adipose tissue (BAT) becomes the favorite target for preventing and treating metabolic diseases because the activated BAT can produce heat and consume energy. The brain, especially the hypothalamus, which secretes Neuropeptide Y (NPY), is speculated to regulate BAT activity. However, whether NPY is involved in BAT activity’s central regulation in humans remains unclear. Thus, it’s essential to explore the relationship between brain glucose metabolism and human BAT activity.

**Methods:**

A controlled study with a large sample of healthy adults used Positron emission tomography/computed tomography (PET/CT) to noninvasively investigate BAT’s activity and brain glucose metabolism *in vivo*. Eighty healthy adults with activated BAT according to the PET/CT scan volunteered to be the BAT positive group, while 80 healthy adults without activated BAT but with the same gender, similar age, and BMI, scanning on the same day, were recruited as the control (BAT negative). We use Statistical parametric mapping (SPM) to analyze the brain image data, Picture Archiving & Communication System (PACS), and PET/CT Viewer software to calculate the semi-quantitative values of brain glucose metabolism and BAT activity. ELISA tested the levels of fasting plasma NPY. The multiple linear regression models were used to analyze the correlation between brain glucose metabolism, the level of NPY, and the BAT activity in the BAT positive group.

**Results:**

(1) Compared with controls, BAT positive group showed significant metabolic decreases mainly in the right Insula (BA13a, BA13b) and the right claustrum (uncorrected P <0.01, adjusted BMI). (2) The three brain regions’ semi-quantitative values in the BAT positive group were significantly lower than the negative group (all P values < 0.05). (3) After adjusting for age, gender, BMI, and outside temperature, there was a negative correlation between brain metabolic values and BAT activity (all P values < 0.05). However, after further adjusting for NPY level, there were no significant differences between the BA13b metabolic values and BAT activity (P>0.05), while the correlation between the BA13a metabolic values and BAT activity still was significant (P< 0.05).

**Conclusions:**

Regional brain glucose metabolism is closely related to healthy adults’ BAT activity, which may be mediated by NPY.

## Introduction

Brown adipose tissue (BAT) has been well recognized as a significant thermogenic tissue to maintain the body temperature in rodents and newborn humans because of its high expression of uncoupling protein 1 (UCP1) (1). Besides, recent studies have proved that BAT can be activated and also present in adults, which are detected by ^18^F-fluorodeoxyglucose (^18^F-FDG) positron emission tomography/computed tomography (PET/CT) (2, 3). Stimulating BAT activity is expected to be a new approach to treat obesity and other metabolic diseases (4, 5), but the precise regulatory mechanism of BAT activity is still unclear.

The central nervous system (CNS) is the core of regulating energy metabolism, which may be related to heat generation and energy consumption of BAT (6), especially the hypothalamus, which is involved in the regulation of BAT activity (7). The hypothalamus is the center of feeding behavior, body temperature regulation, and metabolism control (8). As the region of information integration in the brain, the insular cortex (Insula) regulates temperature, pain, appetite, and energy metabolism, which has a fibrous connection with the hypothalamus (9). The claustrum is a small region subcortical structure and contiguous the insula. Mammal species studies have shown that there are networks between the insula and claustrum, which may influence many aspects of the brain’s function together (10, 11).

Previous invasive methods of studying the structure and function of CNS do not apply to the human body. Emerging molecular functional imaging technology PET/CT is mainly used to diagnose and evaluate tumors, infectious diseases (12), and central nervous system diseases (13), such as lung cancer (14), lymphoma (15), etc. al. Since detection of ^18^F-FDG uptake is the gold standard for reflecting BAT activity, PET/CT imaging has become the most common platform for investigating the human BAT (16). Studies have shown that PET/CT can not only detect the activity of adult BAT (17), but also evaluate the glucose metabolism in specific brain regions (18) and can noninvasively semi-quantitative analyze the brain structure and function. So far, most PET/CT studies focused on oncology patients (19) or with a small sample (20). In several retrospective studies, the prevalence of activated BAT ranged from 1.3% to 6.7%, while our previous retrospective study found that there were 410 of the 31088 subjects (1.32%) identified by PET/CT scanning as activated BAT (21). In our cross-sectional study, the BAT positive rate was 1.77%(105/5945). Moreover, adults with activated BAT were younger, had lower BMI, higher insulin sensitivity, and better lipid profiles than those without activated BAT positive (22).

In view of many factors, such as disease status, age, gender, temperature, and environment, may affect brain metabolism and BAT activity. Therefore, we adopted the ^18^F-FDG PET/CT imaging technology and the Statistical Parametric Mapping (SPM) software, enlarged the sample size, and recruited healthy adults who came to the PET center for the purpose of physical examination in order to precisely explore the relationship between the brain glucose metabolism and healthy adult BAT activity. In addition, this controlled study not only analyzed the relationship between brain glucose metabolism and BAT activity but also verified whether the Neuropeptide Y(NPY), which was secreted by the hypothalamus, was involved in brain glucose metabolism to regulate BAT’s activity. To provide clues and basis for revealing the mechanism of central regulation of BAT activity.

## Materials and Methods

### Subjects and Workflow

**Figure 1:**
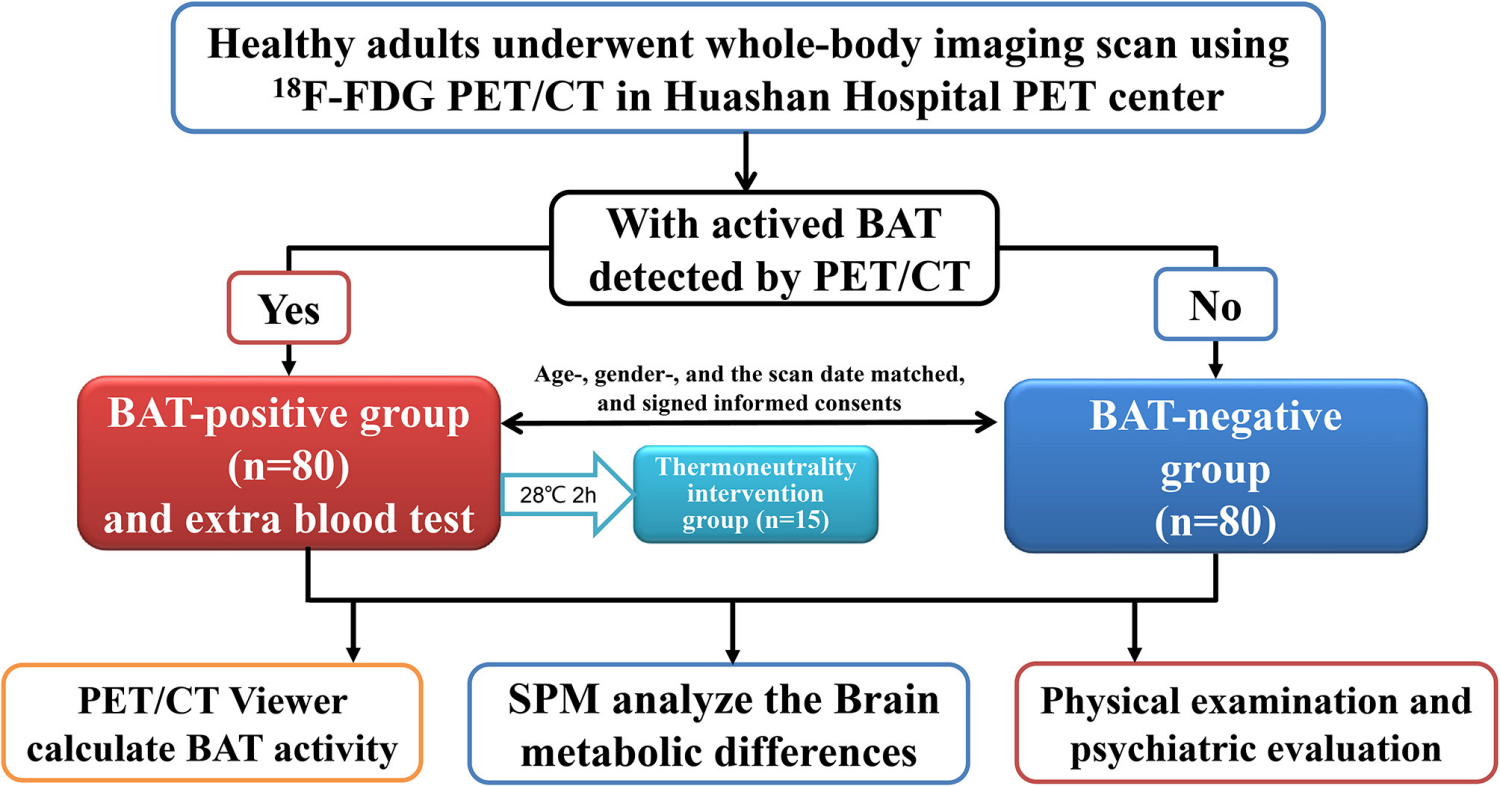
Subjects and Workflow. Flowchart of the PET/CT study: This research is a cross-sectional control study with a large sample. Subjects were divided into two groups according to ^18^F-FDG uptake of BAT detected by PET-CT. Eighty healthy adults with activated BAT detected by PET/CT scan were recruited as the BAT positive group, while each BAT positive subject was matched to a healthy adult without activated BAT detected by PET/CT scan on the same day, with the same gender, and similar age ( ± 5 years), who became the BAT negative control group. There were 15 BAT positive subjects who volunteered to stay in thermoneutrality(28°C, two hours), repeat the PET/CT scan, and take a blood test for the NPY level. Every subject performed the whole-body PET/CT scan, the physical examination, and the blood test.

About six thousands of individuals underwent a whole-body imaging scan using ^18^F-FDG PET/CT in the PET Center of Huashan Hospital Affiliated with Fudan University from September 2009 to March 2011. Eighty healthy adults with activated BAT detected by PET/CT scan were recruited as the BAT positive group after reviewing the medical history, physical examination, and tested fasting blood sample. Each BAT positive subject was matched to a healthy adult without activated BAT detected by PET/CT scan on the same day, with the same gender and similar age ( ± 5 years). Furthermore, in the BAT positive group, there were 15 subjects who volunteered to stay at thermoneutrality (28°C, two hours, in the climate chamber) and then re-examined PET/CT scan to confirm that BAT was undetected within two weeks, and took a fasting blood test for the level of NPY. All subjects were healthy Chinese adults (aged between 20-50 with BMI < 30), and had no history of diabetes, hypertension, heart disease, severe infection, chronic liver or kidney disease, or cancers, and did not take any regular medications (especially adrenergic receptor blockers, antidepressants, or psychiatric drugs, et al.). Pregnant women and subjects with implantable medical electronic devices should be excluded. The workflow of the study is shown in **Figure 1**. The Ethics Committee approved the study design of Huashan Hospital at Fudan University. All of the subjects volunteered for scientific research and signed informed consent. The trial was already registered at Clinical Trials.gov (NCT01387438).

### Medical Assessment and Blood Sample Collection

Demographics and clinical data, including age, gender, body mass index (BMI), medical history, medication utilization, diagnosis, and daily average outdoor temperature, were obtained from all subjects (shown in **Table 1**). The blood samples were drawn in the fasting and resting state, and the fast blood glucose (FBG) levels were measured using an automatic biochemical analyzer (Hitachi 7600, Japan) before the PET/CT scan. The concentrations of NPY in the BAT positive group were determined by enzyme-linked immunosorbent assay (Raybiotech, American) after the PET/CT scan. The other medical assessment, such as the heart rate, systolic blood pressure (SBP), diastolic blood pressure (DBP), was performed on the two groups.

**Table 1:** Characteristics of the subjects with activated BAT (BAT Positive) and without ^18^F-FDG BAT uptake (BAT Negative)*.

	BAT-Positive group (n = 80)	BAT-Negative group (n = 80)	*P* value^†^
**Demographic features**			
Gender (men: women)	20: 60	20: 60	1.000
Age (year)	36.84 ± 6.71	36.90 ± 6.24	0.951
Handedness (R:L:N/A)	78: 1: 1	77: 2: 1	—
Outdoor temperature (°C)	8 (6 – 11)	8 (6 – 11)	1.000
**Clinical Characteristics**			
BMI (Kg/m^2^)	21.06 ± 2.38	21.71 ± 1.83	0.053
SBP (mmHg)	123.13 ± 12.46	122.55 ± 10.38	0.752
DBP (mmHg)	79.79 ± 9.54	81.05 ± 8.62	0.381
Heart rate (beats/min)	72.60 ± 7.34	71.00 ± 6.34	0.142
Fasting glucose (mmol/l)	4.86 ± 0.52	4.82 ± 0.57	0.663

*BAT denotes brown adipose tissue, SBP denotes systolic blood pressure, and DBP denotes diastolic blood pressure. BMI (Body-mass index) is the weight in kilograms divided by the square of the height in meters. The continuous variables of the normal distribution are expressed by mean ± standard deviation, and the continuous variables of non-normal distribution are described by median (quaternary interval P25~P75).

^†^P values were obtained by a two-sided test using two independent sample t­Test.

### PET/CT Scan and Imaging Processing

All subjects fasted for at least 8 hours before performing the whole-body PET/CT scan (PET/CT Biograph 64, SIEMENS) at room temperature, which was maintained at 21~23°C. According to the PET/CT scanning protocol, the PET tracer (^18^F-FDG) was administered intravenously with a dose of 5.55-7.40MBq/kg. Then the subjects rested for 45 minutes until imaging began. After a CT transmission scan for attenuation correction, PET scans were acquired and reconstructed with the three-dimensional (3D) reprojection method. Commonly, transmission and emission modes are used alternately to scan brain images. The peak voltage was 120kV, the electric current was 300mA, the scanning time for each bed is 1.5 minutes, and the collection time is at least 10 minutes. The body’s emission scan was 2 minutes per bed position, and five to six bed positions per subject were needed to cover the BAT areas. Brain PET images were reconstructed by - the ordered subset expectation maximization method and Hanning filtering with matrix 168×168×148 and pixel 2.04mm×2.04mm×1.5mm, giving a transaxial and axial cut-off frequency of 0.5. The Leonardo workstation of Siemens fused the images. Both PET and CT images were reconstructed in transaxial, coronal, and sagittal images with a slice thickness of 3 mm. BAT positive was defined, and BAT activity was calculated according to the standard of Cypess et al. (23): 1) the CT value was adipose tissue (-250 to -50 Hounsfield units), 2) the diameter of the area is greater than 4mm, 3) the maximum of ^18^F-FDG Standardized uptake value (SUV_max_) of BAT exceeds 2.0g/ml. All studies in patients and healthy subjects were performed in a resting state in a quiet and dimly lit room.

### Statistical Analysis

#### Image Data Preprocess

**Figure 2:**
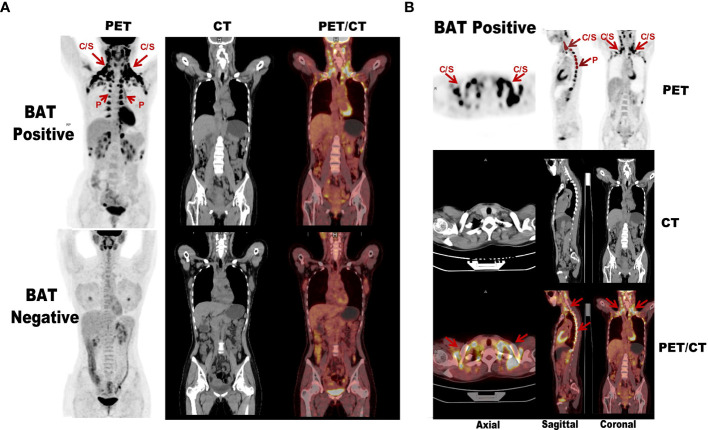
Comparison diagram of subjects with and without activated BAT assessed by ^18^F-FDG PET/CT. **(A)** The above images shows extended BAT uptake of ^18^F-FDG in cervical, supraclavicular, axillary, and paravertebral regions in healthy adult. The below images The below image of the other subject with the same gender, similar age and BMI in whom PET/CT was performed in the same day shows no BAT uptake of ^18^F-FDG in the same regions. **(B)** The subject with activated BAT mainly located in the central axis areas such as the cervical-supraclavicular and paravertebral on the PET maximum intensity projection. “C/S” means cervical/supraclavicular and “P” denotes paravertebral (red arrows).

SPM8 software was used to analyze the locations of brain regions with different metabolic between the BAT positive and negative groups, and also to measure the glucose metabolism of different brain regions and the semi-quantitative value of BAT’s activity. Firstly, PET scans were spatially normalized to Montreal Neurological Institute (MNI) space, by using the default PET template in SPM8 software (Wellcome Department of Imaging Neuroscience, Institute of Neurology, London, UK, Version 8) applied in MATLAB 8.4.0 (Mathworks Inc, Sherborn, MA). Then, in order to increase the signal-to-noise ratio for statistical analysis, the normalized PET scans were smoothed by a Gaussian filter of 10 mm full width at half maximum over a 3D space. Voxel-wise analysis was performed to detect regional differences in mean glucose metabolism between two groups by SPM8 software. A two-sample t-test according to the general linear model at each voxel and the mean signal differences over the whole brain were removed by analysis of covariance in each individual subject. And BMI was included as the covariate. The threshold was set as P<0.01 (uncorrected) over whole brain regions with an extent threshold was empirically chosen to be more than two times of the expected voxels per cluster estimated in the SPM run. Significant regions were localized by Talairach-Daemon software (Research Imaging Center, University of Texas Health Science Center, San Antonio, TX, USA). The SPM map for altered glucose metabolism was overlaid on a standard T1-weighted MRI brain template in stereotaxic space. Next, a spherical volume of interest (VOI), four mm in radius within the image space, centered at the peak voxel of clusters significant in SPM analysis, were constructed to quantify metabolic changes in specific regions (**Figure 2**). We subsequently calculated the individual values of each region as a relative measure of regional cerebral metabolism [VOI value/whole-brain value] using ScAnVP freely available (http://www.feinstein-neuroscience.org at Center for Neuroscience, the Feinstein Institute for Medical Research, Manhasset, NY) in MATLAB.

#### Clinical Data Analysis

SPSS software (Spss Inc., Chicago, IL, USA, version 20.0) was used to analyze the differences in clinical data and semi-quantitative values of brain regions between the BAT positive and negative groups. The Kolmogorov-Smimov test was performed first. Continuous variables of the normal distribution were described by Means ± standard deviation (SD), and Student’s *t*-test was used to compare the differences between the two groups. Data that are not normally distributed are represented as medians (interquartile intervals), and logarithmic conversion is performed before further analysis. Multiple linear regression models were used to analyze the correlation between the semi-quantitative value of brain region and BAT activity and to adjust for possible confounding factors such as age, BMI, gender, outdoor temperature, and the plasma NPY level. All *P* values were two-tailed, and the values less than 0.05 are considered to be statistically significant.

## Results

### Baseline and Characteristics

A total of 80 healthy subjects with detected BAT by PET-CT scan were enrolled into the BAT-positive group, and 80 subjects with well-matched gender (P=1.000), outdoor temperature (P=1.000), and age (P=0.951) and handedness (P=1.000) without detected BAT by PET-CT scan were served as the BAT negative control group. Clinical characteristics between the two groups, such as BMI (21.06 ± 2.38 *vs.* 21.71 ± 1.83, *P* = 0.053), SBP(*P* = 0.752), DBP (*P* = 0.381), heart rate (*P* = 0.234), and FBG (*P* = 0.663) were not statistically different (**Table 1**).

### Differences in Brain Glucose Metabolism Between the BAT Positive Group and the BAT Negative Group

**Figure 3:**
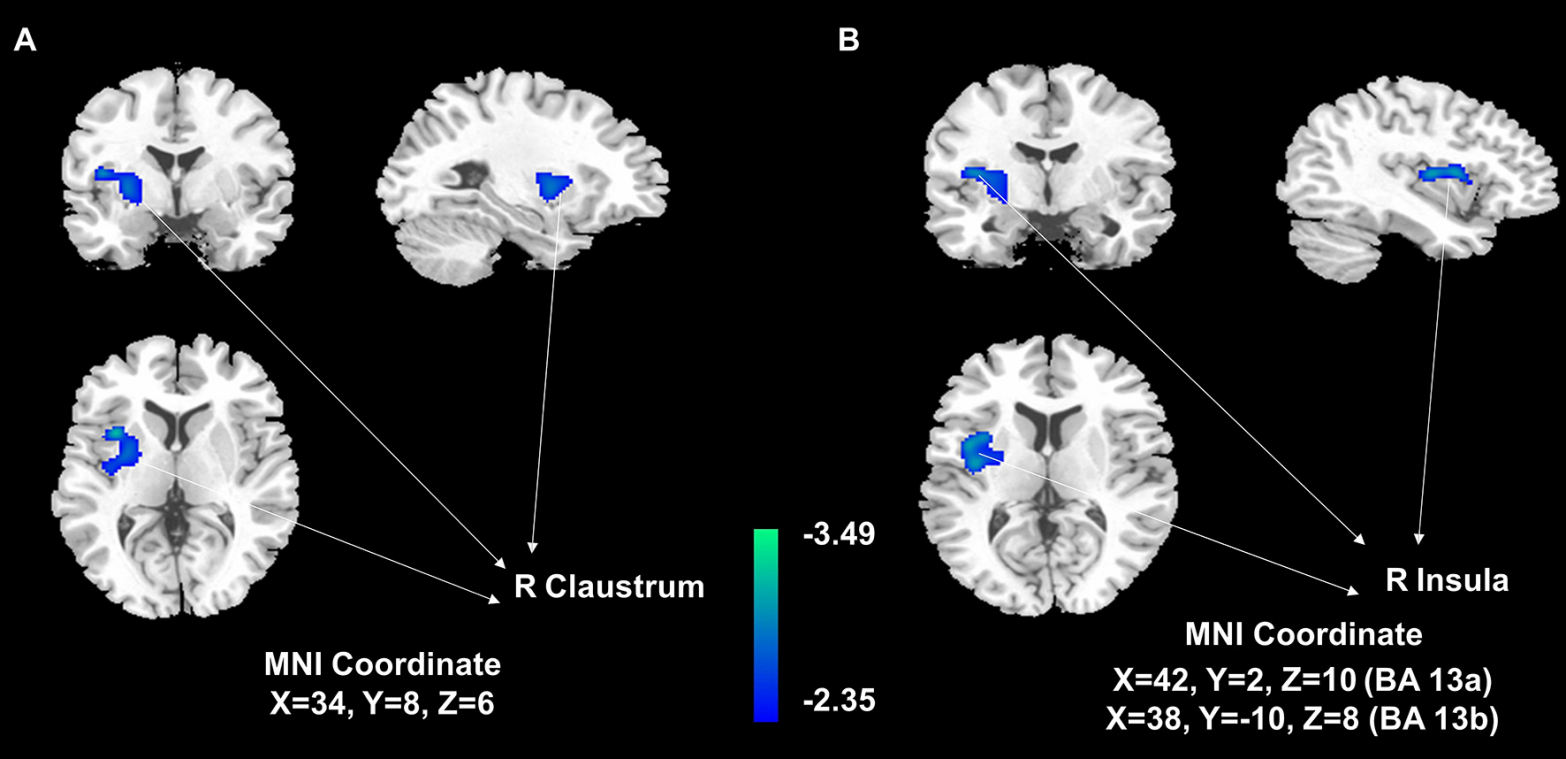
Areas of significant differences of glucose metabolism in subjects with activated BAT, compared with controls. Statistical Parametric Mapping (SPM) results: It is displayed on a T1 template overlaid with magnetic resonance images at the threshold of uncorrected *P*<0. 01, threshold K>321.6, and the gray-scale figures are a T1 structural MRI that are representative of MNI space. The blue areas represent relatively hypometabolic regions of decreased ^18^F-FDG uptake in the right claustrum **(A)** and right Insula **(B)** in BAT-positive subjects than in negative control subjects.

**Figure 4:**
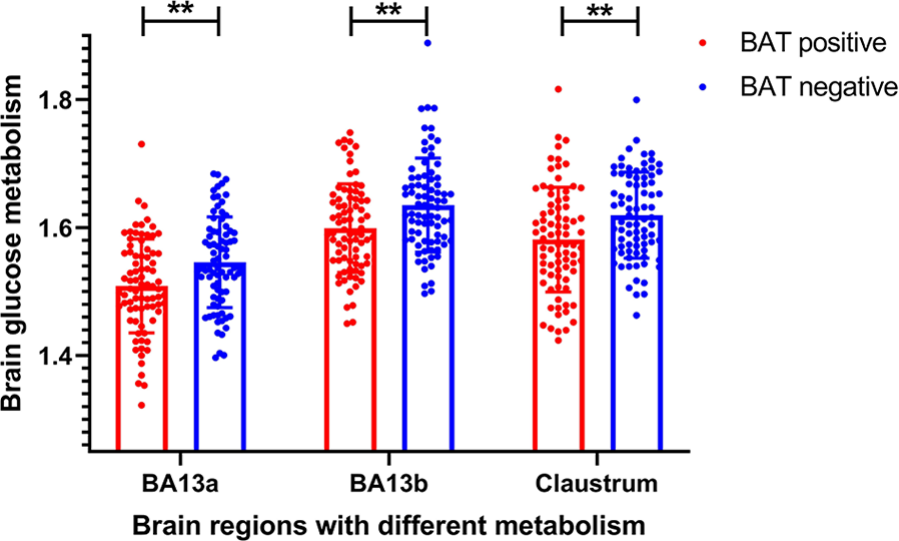
Differences of semi-quantitative metabolic values of brain regions between BAT positive group and BAT negative group. The semi-quantitative metabolic values of three brain region in BAT positive group were significantly lower than these in BAT negative group (BA13_a_ *P* = 0.001; BA13_b_ *P* = 0.002; Right claustrum *P* = 0.002). BA denotes the Brodmann area. *P* values are based on Independent-Samples *t*­Test. ** denotes *P* value < 0.01.

The voxel-wise analysis found that there were differences in three brain regions between the BAT positive group and the BAT negative group (**Figure 3**, and **Table 2**). As shown in **Figure 4**, compared with the BAT negative control group, the local glucose metabolic activities of the right Insula (BA13a, Z =3.08), the right Insula (BA13b, Z =3.05), and the right claustrum (Z =3.16) in the BAT positive group were lower (uncorrected P<0.01, threshold K≥321.6 voxels, adjusting for BMI) (**Table 2**). Then, the semi-quantitative values of glucose metabolism in the three different brain regions were further calculated and analyzed as continuous variables, shown in **Table 3**. Compared with BAT negative controls, the BAT positive subjects’ semi-quantitative metabolic activities of brain regions in the right Insula(BA13a, BA13b) and right claustrum were significantly decreased (**Figure 4** and **Table 3**).

**Table 2:** Areas of significant differences of glucose metabolism in subjects with activated BAT (BAT-positive group), compared with controls (BAT-negative group)*.

Metabolic change	Cluster Size (mm^3^)	T_max_	Z_max_	Coordinates			Brain regions
				x	y	z	
**Decreased**	7864	3.13	3.08	42	2	10	Right Sub-lobar, Insula, Brodmann area 13_a_
	7864	3.10	3.05	38	-10	8	Right Sub-lobar, Insula, Brodmann area 13_b_
	7864	3.21	3.16	34	8	6	Right Claustrum, Brodmann area/

*Coordinates are displayed in MNI standard space. Regions are significant at voxel threshold P < 0.01 (uncorrected, adjusted for BMI), extent threshold =321.6 voxels (2572.8 mm^3^).

**Table 3:** The semi-quantitative values of brain regions with significant metabolic differences between BAT positive group and BAT negative group*.

Brain region	BAT -positive (n = 80)	BAT-negative (n = 80)	*P* value^†^
Right Sub-lobar, Insula (BA13_a_)	1.509 ± 0.008	1.546 ± 0.008	0.001
Right Sub-lobar, Insula (BA13_b_)	1.599 ± 0.008	1.635 ± 0.008	0.002
Right Claustrum	1.581 ± 0.009	1.619 ± 0.008	0.002

*Indicated are mean values ± SE, BA denotes Brodmann area.

^†^P values are based on Independent-Samples t­Test.

### Correlation Analysis of Brain Glucose Metabolism and BAT Activity

**Figure 5:**
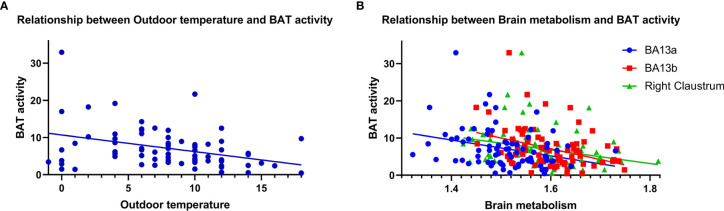
Correlation analysis of brain semi-quantitative metabolism values and average outdoor temperature with BAT activity. BAT activity was negatively correlated with average outdoor temperature [**(A)**, *r* = -0.366, *P* = 0.001] and semi-quantitative values of brain metabolism. [**(B)**, BA13a *r* = -0.293, *P* = 0.008; BA13b *r* = -0.374, *P* = 0.001; right claustrum *r* = -0.279, *P* = 0.012].

As shown in **Figure 5**, there was a negative correlation between the BAT activity and the average outdoor temperature on the examination day in the BAT positive group (*r* = -0.366, *P* = 0.001). And more importantly, the semi-quantitative values of the three different brain regions were both negatively correlated with BAT Activity in the BAT positive group (BA13_a_ *r* = -0.293, *P* = 0.008; BA13_b_ *r* = -0.374, *P* = 0.001; right claustrum *r* = -0.279, *P* = 0.012).

### Multiple Linear Regression Analysis of Brain Glucose Metabolism, NPY Level, and BAT Activity

In order to reduce the interference of confounding factors, multiple linear regression models were used to analyze the correlation between variables in the BAT positive group. The BAT activity was taken as the dependent variable, and the semi-quantitative metabolic values of the three brain regions, as well as the plasma NPY level in the BAT positive group, were analyzed as the independent variable.

**Figure 6:**
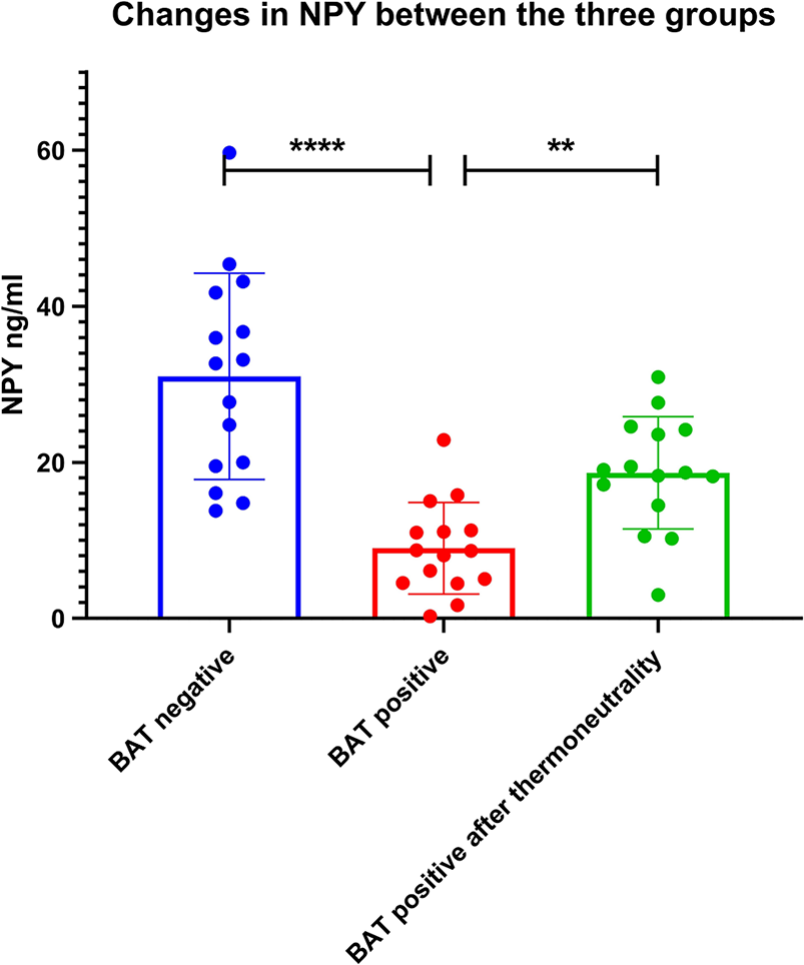
Changes in NPY between the three groups. The NPY levels of 15 BAT positive subjects were lower than their negative controls’ (*P* < 0.001), which also increased after thermoneutrality (*P* = 0.003). ** denotes *P* value < 0.01, **** denotes *P* value < 0.001.

As shown in **Table 4**, after adjusting for age, gender, BMI, and outside temperature in the regression model 1 and 2, the semi-quantitative metabolic values of right insula and claustrum areas were still significantly negatively correlated with BAT activity. While adjusting for the NPY level in the regression model 3, the correlation between the semi-quantitative values of brain metabolism (BA13_a_ and right claustrum) and the BAT activity was statistically negative (*P* = 0.025, *P* = 0.027). It was suggested that the correlation between the other brain region’s semi-quantitative value and BAT activity was affected by NPY levels. However, NPY level was negatively correlated with BAT activity. This negative correlation was still statistically significant even after adjusting for the three brain regions’ semi-quantitative values in the last model (P <0.001), which indicated that the level of NPY was an independent factor affecting BAT activity. The NPY levels of 15 BAT positive subjects were lower than their negative controls’, which also increased after thermoneutrality (**Figure 6**).

**Table 4:** Multiple linear regression models for associations of brain glucose metabolism and NPY with the activity of BAT*.

Independent Variable	Model	β	Standard error of the coefficient	*t*	*ρ*
Region1	1	-22.778	8.380	-2.718	0.008
	2	-22.028	7.812	-2.820	0.006
	3^†^	-15.046	6.585	-2.285	0.025
Region2	1	-30.296	8.353	-3.627	0.001
	2	-29.471	7.754	-3.801	<0.001
	3^†^	-14.261	7.404	-1.926	0.058
Region3	1	-19.221	7.310	-2.630	0.010
	2	-21.880	6.718	-3.257	0.002
	3^†^	-13.256	5.872	-2.257	0.027
Lg(NPY)^‡^	1	-8.331	1.315	-6.334	<0.001
	2	-7.764	1.247	-6.226	<0.001
	3^§^	-6.815	1.343	-5.074	<0.001

*Values of β are regression coefficients; Region 1, 2, 3 denote the semi-quantitative metabolic values of brain regions(BA13a, BA13b, Right Claustrum) respectively; NPY denotes neuropeptide Y; model 1, after adjustment for age, gender, BMI; model 2, after further adjustment for the outside temperature.

^†^Model 3, after further adjustment for NPY.

^‡^We used the logarithmic transformation to transform data to the normal distribution and described them as Lg(X) instead of X.

^§^Model 3, after further adjustment for Region1, Region2, Region3.

## Discussion

This research carried out a controlled study in healthy people with a large sample size to explore BAT activity’s central regulatory mechanism noninvasively and in-vivo and minimizing distractions. Advanced molecular functional imaging PET-CT technology was used to semi-quantitatively analyze the relationship between brain glucose metabolism and BAT activity. Moreover, the plasma NPY level of multiple groups (BAT negative group, BAT positive group, and BAT positive group after thermal neutral intervention) was further detected by Elisa. It provides a new clue to study the central regulatory mechanism of BAT activity.

It is known that the CNS is involved in maintaining energy metabolism and substance metabolism homeostasis, especially the hypothalamus area, which plays an essential role in this process (24). Even though animal studies have shown that knockdown of NPY expression in the dorsomedial hypothalamus could stimulate BAT activity (25), there is no direct evidence that the hypothalamus regulates BAT activity in healthy adults. Our results show apparent metabolic differences in brain regions (such as right insula and claustrum) between BAT positive subjects and their negative controls, but do not show the glucose metabolism values of the hypothalamus region are different between the two groups, which is consistent with the conclusion of the Taiwan study of cancer patients (26). Our research group has also analyzed the changes in brain glucose metabolism in the BAT positive subjects before and after the thermoneutrality intervention, and the metabolic changes in the hypothalamus region were not statistically significant either (18). It may be the hypothalamus region is relatively small, and the PET/CT imaging resolution is limited, so it is difficult to find the area with a difference of less than 2mm. Thus, the possibility of false negatives is likely to exist. The central neuroendocrine regulation is a complex network, and the hypothalamus may play an indirect role through the combination of other fibers or the brain structure.

In this study, SPM8 software and ^18^F-FDG PET/CT related image analysis software were used to further calculate the three brain regions’ semi-quantitative values with metabolic differences and BAT activity. Results show that the semi-quantitative values of the brain regions in the right sub-lobar insula and right claustrum of the BAT positive group were significantly lower than those of the negative group (**Figures 3**, **4** and **Table 3**), which not only analyzed the spatial structure but also semi-quantitatively found that the metabolism of specific brain regions was negatively correlated with BAT activity. These computation results are statistically significant and highly reliable.

Confounding factors such as tumor diseases, age, gender, and temperature can directly influence the glucose metabolism of the human brain and the activity of the BAT (23, 27, 28). Therefore, 160 healthy adults were recruited as this research’s subjects, whether activated BAT detected by PET/CT scan is divided into BAT positive group and negative control group, in accordance with the 1:1 matching of gender and scan date. Demographic characteristics (such as age, gender, hand dominance, and average outdoor temperature) of the two groups were not significantly different, as well as clinical data (such as blood pressure, heart rate, fasting glucose, and BMI) (**Table 1**).

To further reduce the interference of confounding factors, this study establishes the multivariate linear regression models to adjust for age, gender, BMI, and the average outdoor temperature. Despite adjusting for these factors, the semi-quantitative metabolism values of the right insula and claustrum were still negatively correlated with BAT activity. Nevertheless, after further correction of NPY level, only partial right insula and claustrum metabolism values were significantly negatively correlated with BAT activity. These results indicated that the brain glucose metabolism was closely related to BAT activity, which was not affected by age, gender, BMI, and outdoor temperature, but might be affected by the level of NPY.

Although there was no significant metabolic difference in the hypothalamus region between the two groups, in the multiple linear regression models, the level of NPY was consistently negatively correlated with BAT activity after successively adjusting for age, gender, BMI, outdoor mean temperature, and the semi-quantitative values of the three brain regions. The results of this study highly suggested that the level of hypothalamic neuroendocrine factor NPY was closely related to BAT activity and was not affected by other factors. NPY is made up of 36 amino acids, is one of the most critical neuroendocrine factors, mainly secreted by the hypothalamus, stimulates the appetite. While the permeability of the blood-brain barrier is different between individuals, the hypothalamic arcuate nucleus of NPY content is the most abundant. The circulating NPY level is closely related to the central brain source of NPY and can be integrated into the metabolic response of the central and peripheral nervous systems. Our clinical study revealed that under a fasting state, NPY was negatively correlated with BAT activity, and these brain regions might regulate the activity of BAT through the action of NPY. The NPY levels of 15 BAT positive subjects were lower than their negative controls’, which also increased after thermoneutrality intervention. This changing trend of NPY further confirms the negative correlation between NPY and BAT activity.

In this study, the classical Brodmann area (BA) based on the neuroanatomical cellular structure was used to represent the distribution locations of metabolic different brain regions (29). Our study identified the three brain regions of BA13a, BA13b, and right claustrum, which are parts of the right sub-lobar insula and belong to the limbic system. The insula is the only cortex of the human brain hidden deep in the cerebral tissue, in the shape of an inverted triangle, about the size of a “plum”. The insular lobe is involved in many physiological processes such as emotional and psychological regulation, visceral and somatosensory, motion control, etc. (30–32). The insular lobe receives physiological signals (such as temperature, visceral sensation, pain, etc.) that the body senses and then integrates information and transmits it to related structures in the brain. The structure and fiber connections around the insula are the material basis for successfully completing the “bridge” work of the insula. The anterior part of the insula is connected to the anterior lobe of the brain and the posterior portion to the temporal and parietal lobes. In addition, the insular lobe can also be closely associated with the surrounding brain tissue through the superior longitudinal tract, hooklike tract, frontal occipital tract, and other white matter fibers (33, 34). Both the insular lobe (which belongs to the cortical structure) and the hypothalamus (which belongs to the subcortical structure) are components of the limbic system and share a common molecular biomarker, limbic system-associate membrane protein(LAMP). It has a unique function in a specific area and also plays a holistic role through a complex network of peripheral structures, fibrous connections, and loops that interact with other tissues in the brain (35).

As the center of body temperature regulation, the hypothalamus can stimulate BAT activity to produce heat through the sympathetic nervous system, and the insula is the signal receiving station and integration center of the body to the external temperature. Therefore, when the human body is stimulated by ambient temperature, the metabolism of the insula region of the cerebral cortex changes, and the integrated signal is transmitted to the hypothalamus through fiber connections, thereby regulating the thermogenic activity of BAT. However, after the adjustment of outdoor temperature in the multiple linear regression model, it was still found that the metabolism of brain regions was related to BAT activity, suggesting that there were some regulatory mechanisms independent of temperatures, such as mental factors and visceral sensation factors.

There are still some limitations to this study. As a single-center, cross-sectional study, it was observed that glucose metabolism was closely related to BAT activity in some brain regions, but the causal relationship could not be fully explained. Further mechanism studies using transgenic animals are expected. This study focused on the hypothalamic region and NPY, while it is not known that other neuroendocrine factors may be involved in the central regulation of BAT activity. There are still many mysteries in the regulation mechanism of BAT activity, especially in the CNS regulation mechanism. We hope to conduct in-depth research on the mechanism of central regulation of metabolism *in vivo* through micro PET/CT of animals.

## Conclusion

This study revealed that the glucose metabolism in some brain regions (such as the insula-claustrum region) was closely related to BAT activity, and NPY mostly excreted from the hypothalamus may play an essential role in it. This is a new clue added to the central regulation of energy metabolism, which provides valuable experience for opening up new research methodology.

## Data Availability Statement

The data analyzed in this study is subject to the following licenses/restrictions: The data may be related to the privacy of clinical subjects. Requests to access these datasets should be directed to qiongyue_zhang@fudan.edu.cn.

## Ethics Statement

The studies involving human participants were reviewed and approved by the ethics committees of Huashan Hospital, Fudan University in Shanghai. The patients/participants provided their written informed consent to participate in this study.

## Author Contributions

HY, CZ, QZ, YL, and YY designed this study. QZ, QM, YY, WW, QS, BX, JL, and HZ contributed to the recruitment and conducted experiments. QZ and QM wrote the paper. XZ, YF, JL and HZ analyzed data and revised the article. HY, CZ, YL and YG supervised the study. All authors contributed to the article and approved the submitted version.

## Funding

This work was supported by grants from the National Key Research and Development Program of China (2019YFA0801900, 2016YFC1305105), the National Natural Science Foundation of China (81800691, 81670751, 81770840, 81971641, and 81671239), China Postdoctoral Science Foundation (2021M690680), the Shanghai Committee of Science and Technology, China (No. 10JC1401002), the Research project of Shanghai Health Commission (2020YJZX0111), Shanghai Municipal Commission of Health and Family Planning (20144Y0070, 20164Y0041), Clinical Research Plan of SHDC (SHDC2020CR1038B).

## Conflict of Interest

The authors declare that the research was conducted in the absence of any commercial or financial relationships that could be construed as a potential conflict of interest.
